# Experimental Study of Antiatherosclerosis Effects with Hederagenin in Rats

**DOI:** 10.1155/2015/456354

**Published:** 2015-10-19

**Authors:** Su-Hong Lu, Jian-Hua Guan, Yan-Li Huang, Yu-Wei Pan, Wei Yang, Hai Lan, Si Huang, Jing Hu, Guo-Ping Zhao

**Affiliations:** ^1^Medical School, Jinan University, 601 Huangpu Road West, Guangzhou, Guangdong 510632, China; ^2^The First Affiliated Hospital of Jinan University, 613 Huangpu Road West, Guangzhou, Guangdong 510632, China

## Abstract

The research tries to establish Wistar rat's model of atherosclerosis for evaluating the antiatherosclerotic effect of hederagenin and exploring its antiatherosclerosis-related mechanisms. The statistical data have shown that hederagenin exhibits multiple pharmacological activities in the treatment of hyperlipidemia, antiplatelet aggregation, liver protection, and anti-inflammation, indicating that hederagenin may exert a protective effect on vascular walls by improving lipid metabolism disorders and lipid deposition. The results show that hederagenin can correct the imbalance of endothelial function by inhibiting the release of large amounts of iNOS and increasing eNOS contents and inhibits the IKK*β*/NF-*κ*B signaling pathway to reduce the release of IL-6, IFN-*γ*, TNF-*α*, and other inflammatory factors. The experimental results indicated that hederagenin can inhibit or ameliorate the pathological changes associated with AS, displaying an excellent preventive function against AS.

## 1. Introduction

Atherosclerosis (AS) is an inflammatory disease caused by the lesion-like deposition of lipids (primarily cholesterol and cholesterol ester), carbohydrates, and blood components on the vasculature intima or under the intima of the artery and its branches, in addition to the deposition of connective tissues and calcium. AS is accompanied by the migration of medial smooth muscle cells into the intima and the proliferation of these cells, causing intimal thickening and the formation of AS lesions or fibrous fatty plaque lesions. An inflammatory response is always present in AS. Deaths from cardiac and cerebrovascular accidents caused by AS account for the highest disease mortality in humans. Therefore, AS is referred to as the “number one killer” in western countries, and it is one of the most severe cardiovascular diseases that threaten human health. Hence, strategies for achieving early diagnosis and effective interventions for this disease are urgently required. Many studies have been conducted on the prevention and treatment of AS, both domestically and abroad. Most of these studies have investigated the pathogenesis of AS and attempted to delay the progression of the pathological changes associated with AS through drug interventions. Currently, hyperlipidemia is considered the primary factor involved in the occurrence and development of AS. Good control of blood lipids can significantly slow the progression of AS lesions and reduce morbidity and mortality associated with AS-related cardiovascular diseases. There have been drug studies on the prevention and treatment of atherosclerosis conducted to date. Statins can contribute to the prevention of atherosclerosis, but the liver damage they cause after long-term administration is drawing increasing attention [1–3]. Natural herbs present the characteristics of having many available varieties and low toxic side effects. Identifying single active components of natural herbs that can prevent and treat atherosclerosis is a trend in modern pharmaceutical research and development. Hederagenin, with a molecular formula of C_30_H_48_O_4_, is a pentacyclic triterpenoid saponin that is relatively enriched in* Hedera nepalensisvar sinensis* of the Araliaceae* Hedera* L. genus,* Akebia trifoliata* of the Lardizabalaceae, and* Clematis armandii* Franch and* Holboellia fargesii* Reaub of the Ranunculaceae. Studies have shown that hederagenin exhibits multiple pharmacological activities in the treatment of hyperlipidemia, antilipid peroxidation, antiplatelet aggregation, liver protection, antidepression, anti-inflammation, and diuresis [4–12], indicating that hederagenin may exert a protective effect on vascular walls by improving lipid metabolism disorders and lipid deposition. However, there are currently few reports regarding whether hederagenin can protect arteries from AS lesions through its lipid-lowering and anti-inflammatory functions. Therefore, the present study established a Wistar rat atherosclerosis model in which hederagenin was administered as a preventive intervention to evaluate its preventive function against atherosclerosis and to explore the underlying mechanism, providing an experimental basis for clinical drug administration.

## 2. Materials and Methods

### 2.1. Preparation of Drugs

Consider the following: hederagenin (purity 95%), Nanjing Spring and Autumn Biological Engineering Co., Ltd, batch number: 20131020; vitamin D_3_, specifications: 1 mL: 7.5 mg, Shanghai General Pharmaceutical Co., Ltd., batch number: 121123; atorvastatin calcium (Lipitor), specifications: 20 mg ∗ 7 tablets, Pfizer, batch number: H54107; sodium carboxymethyl cellulose (CMC-Na), Shanghai Bohu Biological Technology Co., batch number: 20130513.

### 2.2. Animals and High-Fat Diet

Male, Specific Pathogen-Free- (SPF-) level Wistar rats with weights of 160–200 g, aged 5~6 weeks (provided by the Experimental Animal Center of Southern Medical University, license number: SCKK2011-0015, animal certificate number: 44002100002027), were used in this study. The animals were fed in animal experimental center of Jinan University SPF animal housing management and given free access to water and food; the feeding room temperature was set at 23°C–25°C, and the relative humidity was approximately 7%. After one week of feeding adaptation, the experiments were initiated. The high-fat diet was composed of 3% cholesterol, 0.5% sodium cholate, 0.2% propylthiouracil, 5% sugar, 10% lard, and 81.3% basic fodder, which were mixed well and irradiated with cobalt-60 (radiation dose 25.0 kGy) before feeding.

### 2.3. Experimental Design

#### 2.3.1. Modeling [13–18]

After one week of adaptive feeding with basic rat fodder, 40 quarantined Wistar rats were selected and weighed. The rats were randomly divided into four groups according to a random number table. These groups included a normal group, model group, atorvastatin calcium (Lipitor) group and hederagenin group, with 10 rats in each group. With the exception of the normal group, the rats in all other groups were administered vitamin D_3_ at 600,000 IU/kg/d via intraperitoneal injection, and they were also fed continuously with the high-fat diet. Furthermore, an additional 100,000 IU/kg of vitamin D_3_ was administered to these rats via intraperitoneal injection in the 3rd, 6th, and 9th weeks of the experiments. The rats in the normal group were administered saline through intraperitoneal injection and were fed with normal fodder.

#### 2.3.2. Grouping and Drug Administration

*Doses and Methods*. The rats in each group were administered the corresponding drugs for each intervention. The rats in the hederagenin group were administered hederagenin at 20 mg/kg/d via gavage. The rats in the atorvastatin calcium group were administered atorvastatin calcium tablets at 5 mg/kg/d via gavage. All drugs were prepared as suspension solutions with 0.5% sodium carboxymethyl cellulose (CMC-Na). The rats in the normal control group and the model group were administered an equal amount of 0.5% CMC-Na via gavage continuously for 12 weeks.

### 2.4. Determination of Indicators

At the end of 12 weeks, arteries were collected from the rats to perform HE staining and observe pathological changes under a light microscope and electron microscope. A Zeiss-Axioskop 20 microscope was used to observe the histological changes in HE-stained sections, and an Axiocan HRc camera was employed to obtain micrographs. Leica Qwin Image Processing and Analysis Software was used to analyze AS lesions and the cross-sectional area of the artery lumen and to calculate the relative area of atherosclerosis lesions (the ratio of the lesion area versus the lumen cross-sectional area), which is represented as a percentage (%). Blood lipids, liver lipids, blood rheology, inflammatory factors, and the gene expression and protein expression of eNOS, iNOS, IKK*β*, p-IKK*β*, and NF-*κ*Bp65 in artery tissue were examined.

### 2.5. Statistical Analysis of the Data

The experimental data were analyzed using Social Sciences (SPSS, USA) 16.0 statistical software. Measured data were represented as the mean ± standard deviation (SD), and comparisons of the means between two groups were carried out with one-way ANOVA. When the variance was homogeneous, the SNK test and Tukey test were applied, whereas when the variance was not homogeneous, the T2 test was used and probability value (*P*) less than 0.05 indicated that the difference was statistically significant.

## 3. Results

### 3.1. Observation of Pathological Changes in the Rat Aorta through Light and Electron Microscopy

#### 3.1.1. Light Microscopy Observations of Rat Aorta HE Staining (200x)

**Figure 1:**
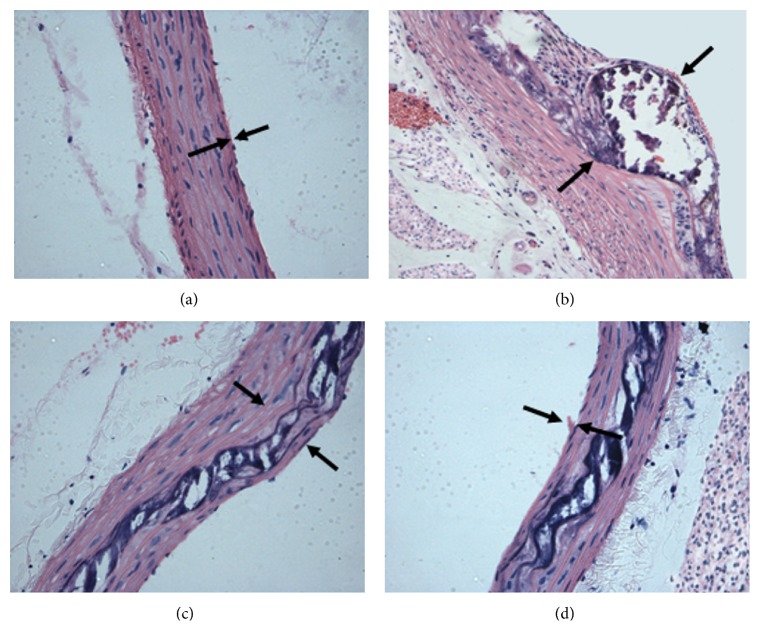
Histological changes of aorta morphology in different groups (HE stain ×200). (a) Normal control group. (b) Model group. (c) Lipitor group. (d) Hederagenin group.

*Normal Control Group*. Aorta morphology was normal; endothelial cells were intact; the intima was smooth; there was no local damage or thickening; the internal elastic lamina was continuous without breaks; the tunica media edge was clear; smooth muscle cells were arranged in an orderly fashion; no inflammatory cell infiltration was observed; and there was no excrescence in the lumen. Model group: significant intimal hyperplasia could be observed in the aorta, which appeared as continuous intimal damage, and a large number of foam cells, cholesterol crystals, intermittent calcification, and unstructured necrotic substances could be observed in the lumen, forming the fibrous cap of AS plaques. The tunica media was thickened, and a large number of smooth muscle cells aggregated and passed through the internal elastic lamina to gather at the tunica intima. Lipitor group: slight intimal thickening of rat aorta, protruding into the lumen, and a thickened tunica media were observed; smooth muscle cells were arranged in a relatively orderly fashion; calcification appeared between the intima and the tunica media; no unstructured necrotic substances were present; and there was a small amount of cholesterol crystal deposition. Hederagenin group: the aorta intima was relatively integral without thickening; only a small amount of damaged intima had detached; the tunica media exhibited intermittent calcification without unstructured necrotic substances; and there was a small amount of cholesterol crystal deposition (Figure 1).

#### 3.1.2. Relative Area of Rat Aorta AS Lesions

**Figure 2:**
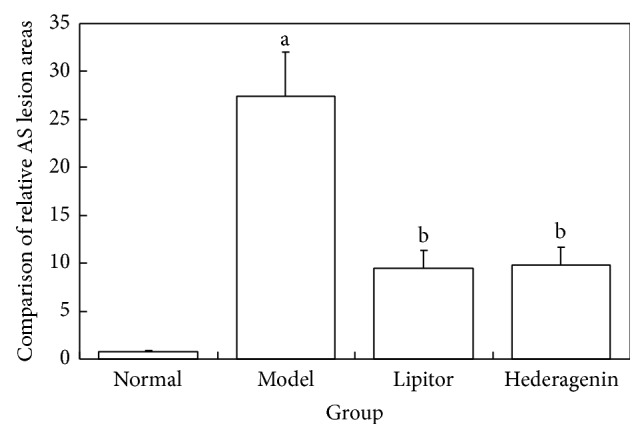
Comparison of relative AS lesion areas in the rat aorta in each group. ^a^ *P* < 0.01 versus normal group; ^b^ *P* < 0.01 versus model group.

*The Experimental Results Demonstrated the Following (Figure 2)*. ① The relative area of model group rat aortic lesions was increased significantly compared with the normal control group, and the difference was statistically significant (*P* < 0.01). ② Compared with the model group, the relative lesion area in the Lipitor group and in the hederagenin group was decreased significantly, and these differences were statistically significant (*P* < 0.01).

#### 3.1.3. Electron Microscopy Observations of the Rat Aorta (12500x)

**Figure 3:**
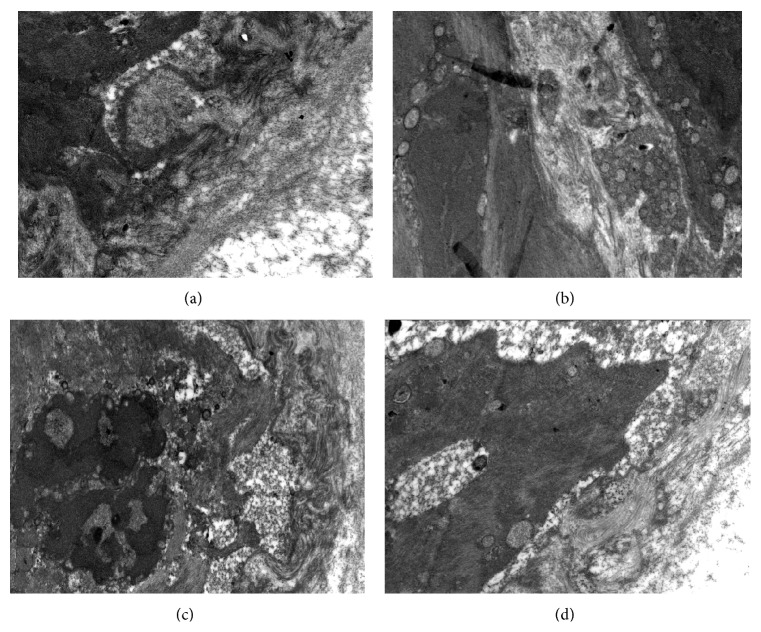
Electron microscopy observations of the rat aorta (12500x). (a) Normal control group. (b) Model group. (c) Lipitor group. (d) Hederagenin group.

*Normal Control Group*. Rat aorta endothelial cells presented a normal morphology; adjacent endothelial cells were closely connected; nuclei were integral; the internal elastic lamina was continuous, with a uniform thickness; no obvious lipid vacuoles or collagen fiber proliferation was observed. Model group: the rat aorta endothelial cells had detached completely; the cytoplasm was not dense; there was a large amount of disorganized lipid deposition. Many collagen fibers could be observed in the intercellular space. Endothelial cell necrosis was observed, where the endothelial cells disappeared and were replaced with cell debris and a large amount of a fiber-like substance. The continuity of the internal elastic lamina was severely damaged, with focal dissolution. There were large amounts of foam cell infiltration, and the number of collagen fibers was increased significantly. Lipitor group: endothelial cells were relatively integral with oval-shaped nuclei; the internal elastic lamina was not uniform, showing thinning with occasional breaks, and thickness was not uniform, with some areas showing a complete loss; the gap between the endothelial cells and the internal elastic lamina increased; collagen fiber deposition and a small number of lipid vacuoles could be observed under the endothelium. Hederagenin group: endothelial cell morphology was relatively normal; local endothelial cell damage could be detected, accompanied by a small amount of smooth muscle cell proliferation; a small number of collagen fibers could be observed in the intercellular space; no endothelial cell necrosis was observed; the structure of the internal elastic lamina was mostly integral; a small amount of collagen fiber deposition could be observed under the endothelium (Figure 3).

### 3.2. Comparison of Rat Serum Lipid Levels, Blood Rheology, and Liver Function in Each Group

#### 3.2.1. Comparison of Rat Serum Lipid Levels in Each Group

**Figure 4:**
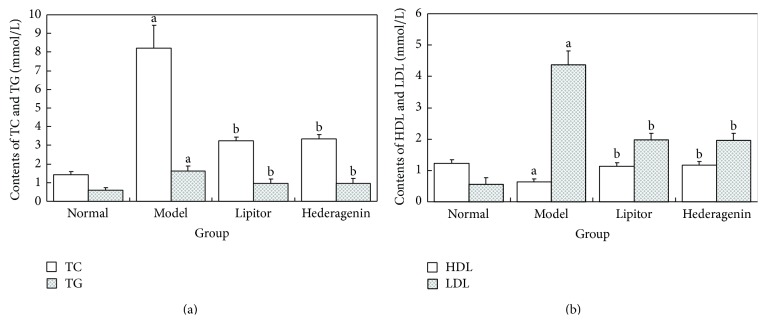
Comparison of rat serum TC, TG, HDL-C, and LDL-C contents in each group. ^a^ *P* < 0.01 versus normal group; ^b^ *P* < 0.01 versus model group.

*Figures 4(a) and 4(b) Showed the Following*. ① Compared with the normal control group, the model group animals exhibited increased TC, TG, and LDL-C levels and decreased HDL-C levels, and these differences were statistically significant (*P* < 0.05 or 0.01). ② Compared with the model group, the Lipitor group and the hederagenin group showed significantly reduced TC, TG, and LDL-C levels and significantly increased HDL-C levels, and these differences were statistically significant (*P* < 0.01).

#### 3.2.2. Comparison of Rat Liver Function in Each Group

**Figure 5:**
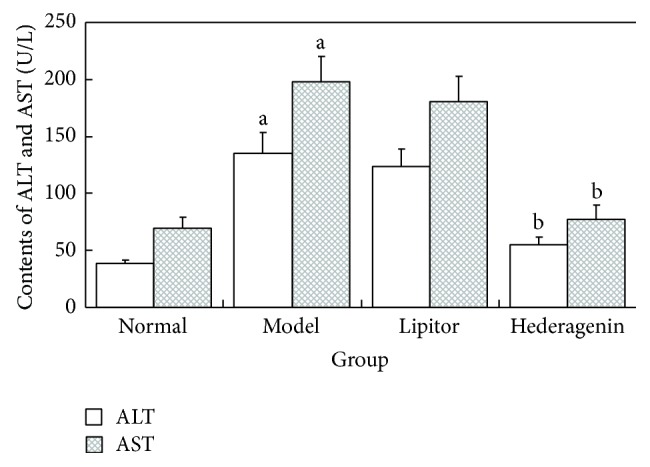
Comparison of rat serum ALT and AST contents. ^a^ *P* < 0.01 versus normal group; ^b^ *P* < 0.01 versus model group.

*The Experimental Results Demonstrated the Following (Figure 5)*. ① Compared with the normal control group, the rats in the model group exhibited significantly increased ALT and AST contents, and these differences were statistically significant (*P* < 0.01). ② Compared with the model group, the hederagenin group showed significantly decreased ALT and AST contents, and these differences were statistically significant (*P* < 0.01). ③ Compared with the model group, the Lipitor group exhibited significantly increased ALT and AST contents, though these differences were not statistically significant (*P* > 0.05).

#### 3.2.3. Comparison of Rat Hemorheological Parameters in Each Group

*The Experimental Results Demonstrated the Following (Table 1)*. ① Compared with the normal control group, rat hemorheological parameters were significantly increased in the model group, and these differences were statistically significant (*P* < 0.01). ② Compared with the model group, rat hemorheological parameters in the Lipitor and hederagenin groups were decreased, and these differences were statistically significant (*P* < 0.01). ③ Compared with the Lipitor group, the hederagenin group exhibited an improved high shear whole-blood viscosity index, and this difference was statistically significant (*P* < 0.05).

### 3.3. Comparison of Rat Aortic Endothelial Function in Each Group

#### 3.3.1. Comparison of Rat Serum NO and ET-1 Contents in Each Group

**Figure 6:**
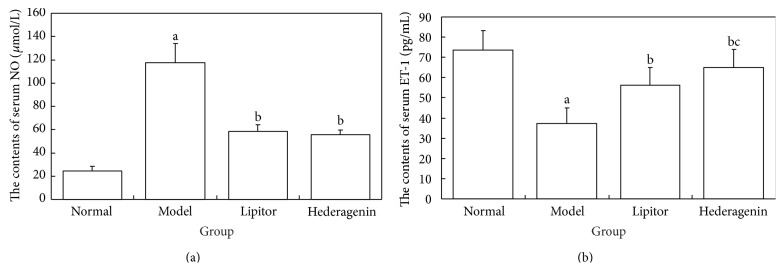
Comparison of rat serum NO and ET-1 contents. ^a^ *P* < 0.01 versus normal group; ^b^ *P* < 0.01 versus model group; ^c^ *P* < 0.05 versus atorvastatin calcium group.

*Figures 6(a) and 6(b) Showed the Following*. ① Compared with the normal control group, the contents of NO significantly increased while ET-1 significantly decreased in the model group and these differences were statistically significant (*P* < 0.01). ② Compared with the model group, the contents of NO significantly decreased while ET-1 significantly increased in the Lipitor and hederagenin groups and these differences were statistically significant (*P* < 0.01). ③ Compared with the Lipitor group, the hederagenin group exhibited significantly increased ET-1 contents, and these differences were statistically significant (*P* > 0.05).

#### 3.3.2. Comparison of Rat Aortic iNOS and eNOS Protein Expression Levels

**Figure 7:**
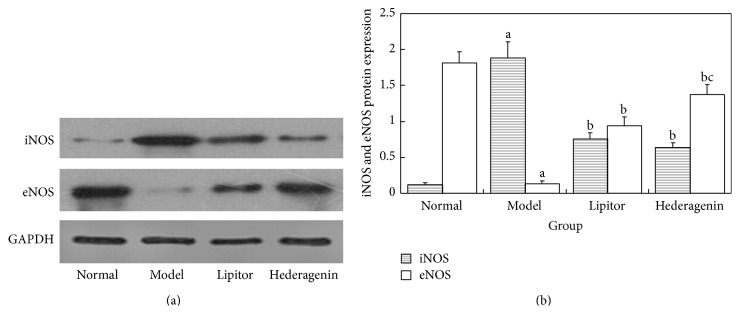
Comparison of rat aortic iNOS and eNOS protein expression levels. ^a^ *P* < 0.01 versus normal group; ^b^ *P* < 0.01 versus model group; ^c^ *P* < 0.05 versus atorvastatin calcium group.

*Figures 7(a) and 7(b) Showed the Following*. ① Compared with the normal control group, the contents of iNOS significantly increased while eNOS significantly decreased in the model group, and these differences were statistically significant (*P* < 0.01). ② Compared with the model group, the contents of iNOS significantly decreased while eNOS significantly increased in the Lipitor and hederagenin groups, and these differences were statistically significant (*P* < 0.01). ③ Compared with the Lipitor group, the hederagenin group exhibited significantly increased eNOS contents, and these differences were statistically significant (*P* > 0.05).

### 3.4. Comparison of the IKK*β*/NF-*κ*B Signaling Pathway and Relevant Inflammatory Factors in the Rat Aorta in Each Group

#### 3.4.1. Contents of the Inflammatory Cytokines IL-6, IFN-*γ*, and TNF-*α* in the Rat Aorta

**Figure 8:**
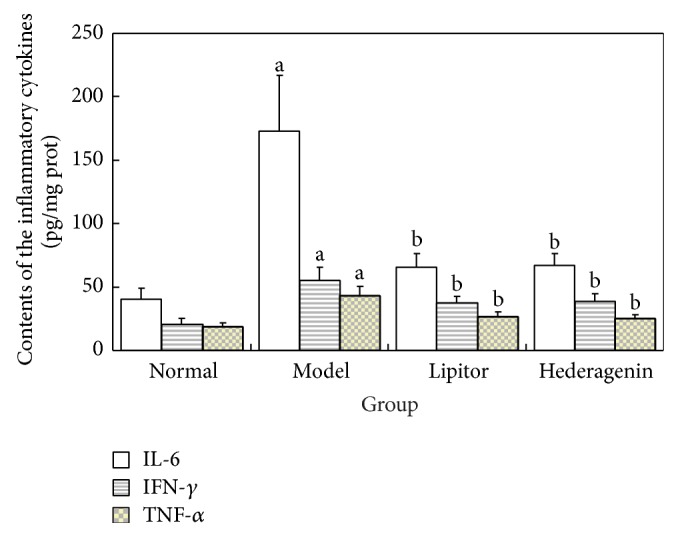
Contents of the inflammatory cytokines IL-6, IFN-*γ*, and TNF-*α* in the aorta. ^a^ *P* < 0.01 versus normal group; ^b^ *P* < 0.01 versus model group.

*The Experimental Results Demonstrated the Following (Figure 8)*. ① Compared with the normal control group, the contents of IL-6, IFN-*γ*, and TNF-*α* significantly increased in the model group, and these differences were statistically significant (*P* < 0.01). ② Compared with the model group, the contents of IL-6, IFN-*γ*, and TNF-*α* significantly decreased in the Lipitor and hederagenin groups, and these differences were statistically significant (*P* < 0.01).

#### 3.4.2. Relative Expression Levels of IKK*β* and NF-*κ*B mRNA in Rat Aortic Tissue in Each Group

**Figure 9:**
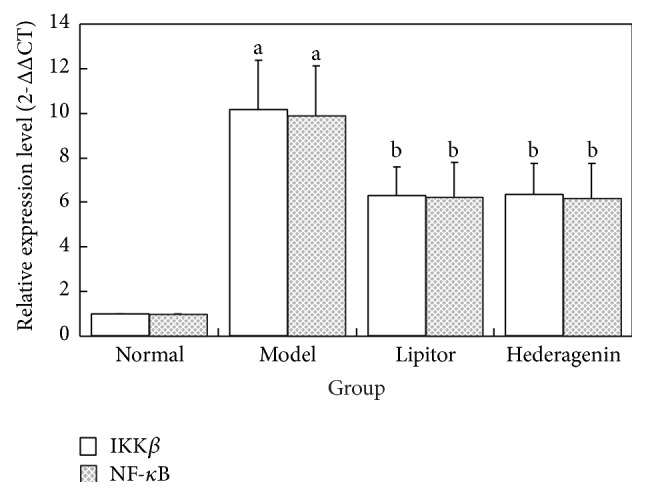
Relative expression level of IKK*β* and NF-*κ*B mRNA in the rat aorta. ^a^ *P* < 0.01 versus normal group; ^b^ *P* < 0.01 versus model group.

*The Experimental Results Demonstrated the Following (Figure 9)*. ① Compared with the normal control group, the model group animals exhibited increased gene expression levels of IKK*β* and NF-*κ*B, and these differences were statistically significant (*P* < 0.01). ② Compared with the model group, the Lipitor group and the hederagenin group showed significantly reduced gene expression levels of IKK*β* and NF-*κ*B, and these differences were statistically significant (*P* < 0.01).

#### 3.4.3. The Expression of Cytoplasmic IKK*β*, p-IKK*β*, and Nuclear NF-*κ*Bp65 Proteins in the Rat Aorta

**Figure 10:**
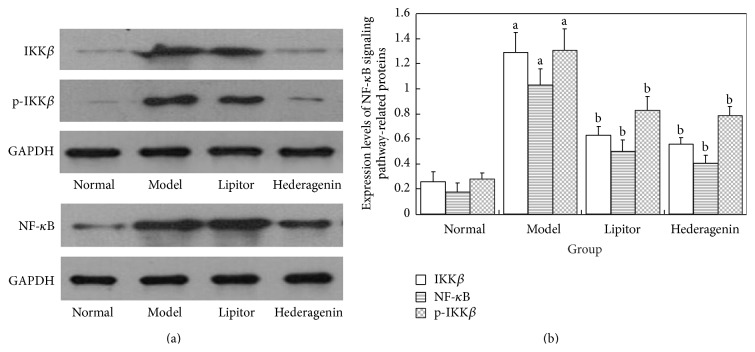
The expression levels of NF-*κ*B signaling pathway-related proteins. ^a^ *P* < 0.01 versus normal group; ^b^ *P* < 0.01 versus model group.

*Figures 10(a) and 10(b) Showed the Following*. ① Compared with the normal control group, the model group animals exhibited increased protein content of IKK*β*, p-IKK*β*, and NF-*κ*B, and these differences were statistically significant (*P* < 0.01). ② Compared with the model group, the Lipitor group and the hederagenin group showed significantly reduced protein content of IKK*β*, p-IKK*β*, and NF-*κ*B, and these differences were statistically significant (*P* < 0.01).

## 4. Discussion

Atherosclerosis (AS) is a complicated pathological process resulting from interactions between various pathways and factors. The theory of AS pathogenesis primarily involves thrombosis theory, lipid filtration theory, endothelial dysfunction theory, oxidation stress theory, and inflammatory response theory. These theories correspondingly explain different aspects of the pathogenesis and progression of atherosclerosis. An increasing number of studies have shown that atherosclerosis is an inflammatory disease, and the inflammatory response is present throughout the process of the pathogenesis and progression of AS. During the early stage of atherosclerosis, when unstable plaques break, there are continuous activation and amplification of inflammation. Therefore, the early identification of unstable AS plaques, detecting sensitive and specific serological markers of these plaques and associated inflammation targets, in addition to reducing and blocking atherosclerosis and other vascular events through early anti-inflammation treatment, represent current research hot spots that will continue to direct future research.

Hyperlipidemia and hemorheological abnormalities usually occur at the same time and promote each other's occurrence. Epidemiological studies have shown that, as an initiation factor of atherosclerosis, hyperlipidemia, accompanied by hemorheological abnormalities, is generally a risk factor for atherosclerosis. Studies have demonstrated that an increase of serum cholesterol levels is positively correlated with the occurrence of AS and can lead to abnormal plasma lipoproteins, thereby inducing artery wall lesions. Lipoproteins are the major form of lipids that exists in human plasma. Low density lipoprotein (LDL) plays an important role in the pathogenesis of atherosclerosis and is considered the major risk factor for atherosclerosis. Drug intervention experiments confirmed that lowering LDL levels can significantly reduce the risk of the occurrence of cardiovascular diseases for hypercholesterolemia patients and can also benefit patients with a normal level of LDL [19]. Studies have shown that one risk factor that causes AS is an overly low level of high density lipoprotein (HDL). HDL can carry out reverse transport of cholesterol to the liver for processing to lower body cholesterol levels, and HDL therefore exhibits an anti-AS function [20, 21]. Studies have shown that every stage of atherosclerosis is accompanied by endothelial dysfunction, and the occurrence of many coronary events is closely related to coronary artery endothelial dysfunction. Endothelial dysfunction is an early event in the occurrence of atherosclerosis, and all of the risk factors that cause atherosclerosis can also cause coronary artery endothelial dysfunction. Damage to the endothelium not only is the initiating step in the occurrence of AS but also serves as a clinical sign of AS disease, which plays an independent role in predicting the prognosis of AS [22–29].

AS is an inflammatory disease, and the inflammatory response is observed throughout the process of the occurrence and progression of AS [30, 31]. Studies have shown that the IKK/I*κ*B/NF-*κ*B signaling pathway plays a key role in the occurrence of AS. Throughout the course of AS, NF-*κ*B is involved in multiple signaling pathways in the inflammatory process. The body's inflammatory response is not separable from the participation of various molecules [32], including IL-6, IL-8, IFN-*γ*, TNF-*α*, intercellular adhesion molecule-1 (ICAM-1), and vascular cell adhesion molecule-1 (VCAM-1), and a prerequisite for the activation of these molecules is activation of NF-*κ*B. In other words, a prerequisite for the inflammatory response is the activation of NF-*κ*B, and the inflammatory response is a prerequisite for AS occurrence [33–36]. As an important cytokine in the progression of AS, IL-6 was recently found to be positively correlated with atherosclerosis. The development of AS lesions is a slow and complicated process related to the inflammatory response. IL-6 represents the origin of the inflammatory response cascade, playing an extremely significant mediating function [37, 38]. IFN-*γ* is a cytokine that was recently found to be positively correlated with atherosclerosis. IFN-*γ* acts on multiple types of cells in AS plaques, regulating the expression of cytokines and cytokine receptors in these cells as well as the proliferation and apoptosis of the cells to promote the formation of AS plaques. Studies have shown that IFN-*γ* is a pro-AS cytokine. Macrophages and smooth muscle cells, which are found in AS plaques, show lipid accumulation and express the IFN-*γ* receptor [39–42]. Therefore, NF-*κ*B activation leads to overexpression of inflammation-related factors, resulting in the inflammatory response [43]. Meanwhile, the increased production and release of inflammatory mediators and cytokines further activate NF-*κ*B, resulting in continuous amplification of the initial inflammation signal and even loss of control of the inflammation response, eventually leading to AS.

Thus, in the present study, we explored and evaluated the antiatherosclerosis function of hederagenin by establishing a Wistar rat AS model, and we analyzed the antiatherosclerosis mechanism of hederagenin from the perspective of lipid metabolism disorders, liver function, blood rheology, endothelial function, and inflammation signaling pathways. Studies have shown that, in AS rat models induced by a high-lipid diet plus VD_3_, hederagenin can effectively reduce serum lipid, ALT, and AST levels, in addition to improving liver function, relieving high blood coagulation, and slowing blood flow and stasis by improving blood rheology. Hederagenin can correct the imbalance of endothelial function by inhibiting the release of large amounts of iNOS and increasing eNOS contents. Hederagenin also inhibits the IKK*β*/NF-*κ*B signaling pathway to reduce the release of IL-6, IFN-*γ*, TNF-*α*, and other inflammatory factors.

## 5. Conclusion

In conclusion, the experimental results showed that hederagenin can inhibit or ameliorate the pathological changes associated with AS, displaying an excellent preventive function against AS. The mechanism of hederagenin action may be related to the regulation of lipid metabolism disorders, protection of liver function, improvement of blood rheology, regulation of endothelial dysfunction, and inhibition of the IKK*β*/NF-*κ*B signaling pathway, thereby reducing the amplification cascade of the inflammatory response, to reduce the release of IL-6, IFN-*γ*, TNF-*α*, and other inflammatory factors. Further study is needed to find out whether there are some other signal transduction pathways involved in the course.

## Figures and Tables

**Table 1:** Comparison of rat hemorheological parameters in each group. ^a^ *P* < 0.01 versus normal group; ^b^ *P* < 0.01 versus model group; ^c^ *P* < 0.05 versus atorvastatin calcium group.

Group	Whole-blood viscosity/mpa·s				Hematocrit	Erythrocyte aggregation index	Platelet aggregation rate/%
	Low shear	Mid shear	High shear	Viscosity			
Normal	10.76 ± 0.83	7.15 ± 0.72	5.18 ± 0.42	1.28 ± 0.12	0.41 ± 0.04	5.12 ± 0.33	39.22 ± 3.69
Model	19.23 ± 2.51^a^	9.66 ± 0.89^a^	7.33 ± 0.76^a^	1.81 ± 0.23^a^	0.69 ± 0.12^a^	6.51 ± 0.87^a^	59.03 ± 5.17^a^
Lipitor	12.64 ± 0.97^b^	7.32 ± 0.83^b^	6.26 ± 0.58^b^	1.32 ± 0.15^b^	0.52 ± 0.05^b^	5.28 ± 0.39^b^	46.61 ± 4.09^b^
Hederagenin	12.58 ± 0.89^b^	7.27 ± 0.71^b^	5.66 ± 0.53^bc^	1.37 ± 0.14^b^	0.53 ± 0.03^b^	5.37 ± 0.45^b^	46.38 ± 4.19^b^
